# Outcome of Percutaneous Release for Trigger Digits in Diabetic and Non-diabetic Patients

**DOI:** 10.7759/cureus.4585

**Published:** 2019-05-02

**Authors:** Adeel A Siddiqui, Irfan Muhammad Rajput, Mariyam Adeel

**Affiliations:** 1 Orthopaedic Surgery, Dow University of Health Sciences, Karachi, PAK; 2 Orthopaedics, Dow University of Health Sciences, Karachi, PAK

**Keywords:** percutaneous finger release, hand surgery, stenosing tenosynovitis, orthopedics, surgical complications, trigger finger, diabetes, outcome, trigger digit, complications

## Abstract

Introduction

Trigger finger (TF) is a common cause of hand pain, swelling, and limited motion. It is common in women and in the thumb. Diabetes mellitus (DM) increases the risk of TF. Individuals with DM who develop TF are resistant to both medical and surgical interventions. The aim of this study is to compare the outcomes of percutaneous trigger release in diabetic and nondiabetic patients.

Methods

Fifty diabetic and 50 non-diabetic patients with a clinical diagnosis of TF were included after informed consent. Percutaneous trigger release was performed in all of them. Follow-ups for pain and/or neurovascular complications were taken after one week, one month, and six months. Data were entered and analyzed using SPSS v. 22 (IBM Corp., Armonk, NY, US).

Results

In the diabetic group, 86% of patients had TF of grade III or above and in the non-diabetic group, 76% of patients had TF of grade III or above. At the one-week follow-up, 79.2% diabetic patients still had mild to severe pain and 60.4% non-diabetic patients had mild to severe pain. By one month, 40% patients in the diabetic group still reported mild to moderate pain, however, all patients in the non-diabetic group reported no pain. By six months, nine (20%) diabetic patients reported mild pain. There was no incidence of infection or neurovascular damage at any follow-up in the non-diabetic group, and in the diabetic group, 4.2% of patients had an infection on the one-week follow-up.

Conclusion

Percutaneous trigger finger release is a safe, reliable, time-saving, and cost-effective procedure for the management of trigger finger in both diabetic and non-diabetic patients.

## Introduction

Trigger finger (TF) or stenosing tenosynovitis is frequently witnessed in general practice. It is a common cause of swelling, pain, limited range of motion, and disability of the hand. TF is common in middle-aged, healthy women. It is most common in the flexor tendons of the thumb, however, it may occur in any digit [1]. The underlying triggering mechanism is induced by the thickening of the A1 pulley, which causes the entrapment of the flexor tendon [2]. The incidence of TF is 28 cases per 100 000 population per annum, with a lifetime risk of 2.6% in the general population [3].

Primary TF is idiopathic and more common than secondary TF, which is common in chronic medical conditions such as gouty and rheumatoid arthritis, kidney diseases, and diabetes mellitus (DM) [1]. The lifetime risk of TF in patients with diabetes mellitus (DM) is 10% [4].

TF presents with popping or painful catching of the flexor tendons when flexing or extending the affected digit. As TF progresses, the digit might become locked in flexion and may need passive or active correction for full range of motion. Over time, patient reluctance due to pain and consequent guarding restricts tendon motion and may secondarily lead to fixed deformity at the proximal interphalangeal (PIP) joint in the form of contractures [5]. There are various modalities for the management of trigger finger. These include local corticosteroid injections, splintage, hydrotherapy, analgesics, and percutaneous release. Open surgery is only recommended when all of these fail [5]. Percutaneous release of the triggering finger has shown promising results. It provides earlier functional recovery and high patient satisfaction [6], and it’s a rapid and cost-effective procedure [7].

Elsayed, in his study, reported 97% of cases with excellent to good results and only one failure. There was no incidence of infection or nerve or tendon injury [8]. Similarly, Ragoowansi et al. also reported excellent to good results in 94% of cases managed with percutaneous release of trigger finger [9]. In a study with comparison of short and long-term outcomes in patients with and without DM, pain in the short term was equally common in both groups, and in the long term, pain was more common in the DM group [10]. The aim of this study is to compare the functional outcome in diabetic and non-diabetic patients with trigger finger managed with percutaneous release.

## Materials and methods

It was a prospective observational study conducted at Dr. Ruth Pfau Hospital, Karachi, for six months from May 1 to November 30, 2017. Data were collected using a self-structured proforma. Patients were recruited from the orthopedic consulting clinics based on a clinical diagnosis of trigger finger, with symptoms including pain, catching, and stiffness of the affected digit. The purpose, procedure, risks, and benefits of the study were explained to the patients and formal written consent was taken. Patients who refused to consent for the procedure, patients who presented with recurrent trigger finger on the same/another digit, and patients who were on anticoagulants were excluded from the study.

Triggering was classified into four grades according to severity (Modified Quinnell Grading System). In grade I, there is pain and tenderness over the A1 pulley with no entrapment or catching during the examination. In grade 2, there is a visible entrapment but the patient is able to extend the finger actively. In grade 3, entrapment needs passive extension (grade 3A) or causes the inability to actively flex the finger (grade 3B). In grade 4, entrapment causes fixed flexion contracture of the finger [1].

Percutaneous trigger release was performed with local anesthesia using 1.5-3 cc of 2% xylocaine and the A1 pulley was released using a 19-gauge needle based on Eastwood's technique [11]. Adhesive dressings were applied and the patients were allowed to immediately resume their daily activities. Icing packs were advised for two-three days post-procedure. Patients were followed up after one week, the second follow-up was after one month of the procedure, and the third follow-up was after six months of the procedure.

At the start of the study, 50 patients were recruited for the procedure in both the diabetic and non-diabetic groups. In the diabetic group, two patients were lost on the first follow-up (after one week) and another three patients were lost on the second follow-up visit (after one month). In the non-diabetic group, seven patients were lost on the first follow-up (after one week) and another four patients were lost on the second follow-up visit (after one month). No other patients were lost at the six-month follow-up.

Pain was assessed on each follow-up visit using the visual analog scale (VAS) [12]. It assesses pain on a 10-item visual scale, where 0 is no pain, 1-2 is mild pain, 3-6 is moderate pain, and 7-10 is severe pain. The complications of the procedure, including neurovascular block and local infection, were recorded on each follow-up visit.

Data were entered and analyzed using SPSS v. 22 (IBM Corp., Armonk, NY, US). Frequency and percentages were calculated for categorical data and mean and standard deviation (SD) were calculated for continuous variables.

## Results

There were more women in the diabetic group (50% vs. 48%) and more men in the non-diabetic group (76% vs. 24%). The mean age of diabetic patients was more than that of non-diabetic patients. In the diabetic group, 56% reported a history of trauma and 82% had HbA1c more than 7%. The demographic and clinical characteristics of diabetic and non-diabetic patients, at baseline, are compared in Table 1.

**Table 1:** Baseline demographic and clinical characteristics of diabetic and non-diabetic patients Abbreviations: SD, standard deviation

Patient characteristics	Frequency (%)	
	Diabetics (n=50)	Non-diabetics (n=50)
Gender		
Male	24 (48%)	38 (76%)
Female	26 (50%)	12 (24%)
Age		
18-45 years	19 (38%)	23 (46%)
> 45 years	31 (62%)	27 (54%)
Mean ± SD	41.58 ± 11.49	38.17 ± 8.87
Trauma history		
Yes	28 (56%)	17 (34%)
No	22 (44%)	33 (66%)
Duration of diabetes		
< 5 years	11 (22%)	----
> 5 years	39 (78%)	----
Mean ± SD	6.47 ± 3.55	----
HbA1c		
< 7%	9 (18%)	----
> 7%	41 (82%)	----
Mean ± SD	8.57 ± 1.78	----
Dominant hand		
Right	46 (92%)	41 (82%)
Left	4 (8%)	9 (12%)
Hand involved		
Right	29 (58%)	20 (40%)
Left	21 (42%)	30 (60%)
Digits involved		
Thumb	27 (54%)	23 (46%)
Index	11 (22%)	18 (36%)
Middle	7 (14%)	3 (6%)
Ring	5 (10%)	6 (12%)

Disease assessment, pain, and other complications at each visit are shown in Table 2. In the diabetic group, there were 43/50 (86%) patients who had TF of grade III or above and in the non-diabetic group, 38/50 (76%) patients had TF of grade III or above. At the one-week follow-up, 38/48 (79.2%) diabetic patients still had mild to severe pain and 26/43 (60.4%) non-diabetic patients had mild to severe pain. By one month, 18/45 (40%) patients in the diabetic group still reported mild to moderate pain, however, all patients in the non-diabetic group reported no pain. There was no incidence of infection or neurovascular damage at any follow-up in the non-diabetic group and in the diabetic group, only 2/48 (4.2%) patients had an infection on the one-week follow-up (Table 2).

**Table 2:** Severity of TF, pain, and other complications in diabetic and non-diabetic patients Abbreviations: VAS, Visual Analogue Scale.

Assessment of outcome	Day 0 (before procedure)		One week of procedure		One month of procedure		Six month of procedure	
	Diabetics (n=50)	Non-diabetics (n=50)	Diabetics (n=48)	Non-diabetics (n=43)	Diabetics (n=45)	Non-diabetics (n=39)	Diabetics (n=45)	Non-diabetics (n=39)
Modified Quinnell System for Trigger Finger grading								
Grade I Normal movement, No Pain	3 (6%)	4 (8%)	7 (14.5%)	16 (37.2%)	17 (37.8%)	28 (71.8%)	39 (86.7%)	38 (97.5%)
Grade II Normal movement, occasional pain	4 (8%)	8 (16%)	16 (33.3%)	14 (32.5%)	23 (51.1%)	11 (28.2%)	6 (13.3)	1 (2.5%)
Grade III Uneven movement	14 (28%)	19 (38%)	23 (47.9%)	13 (30.2%)	5 (11.1%)	--	--	--
Grade IV Intermittent locking, actively correctable	18 (36%)	13 (26%)	2 (4.2%)	--	--	--	--	--
Grade V Locking only passively correctable	11 (22%)	6 (12%)	--	--	--	--	--	--
VAS Scale for pain assessment								
No pain	5 (10%)	5 (10%)	10 (20.8%)	17 (39.5%)	27 (60%)	39 (100%)	36 (80.0%)	39 (100%)
Mild pain	11 (22%)	9 (18%)	20 (41.6%)	20 (46.5%)	15 (33.3%)	--	9 (20.0%)	--
Moderate pain	24 (48%)	20 (40%)	11 (23%)	6 (13.9%)	3 (6.7%)	--	--	--
Severe pain	10 (20%)	16 (32%)	7 (14.5%)	--	--	--	--	--
Complications of the procedure								
Superficial wound Infection	--	--	2 (4.2%)	--	--	--	--	--
Neurapraxia	--	--	--	--	--	--	--	--
Vascular injury	--	--	--	--	--	--	--	--
Others	--	--	--	--	--	--	--	--

Infection in both patients of the diabetic group was managed successfully with empirical antibiotic agents. None of the patients in either group presented with a recurrent trigger during the follow-up period and the procedure did not fail in any patient; a re-procedure was not required.

**Figure 1:**
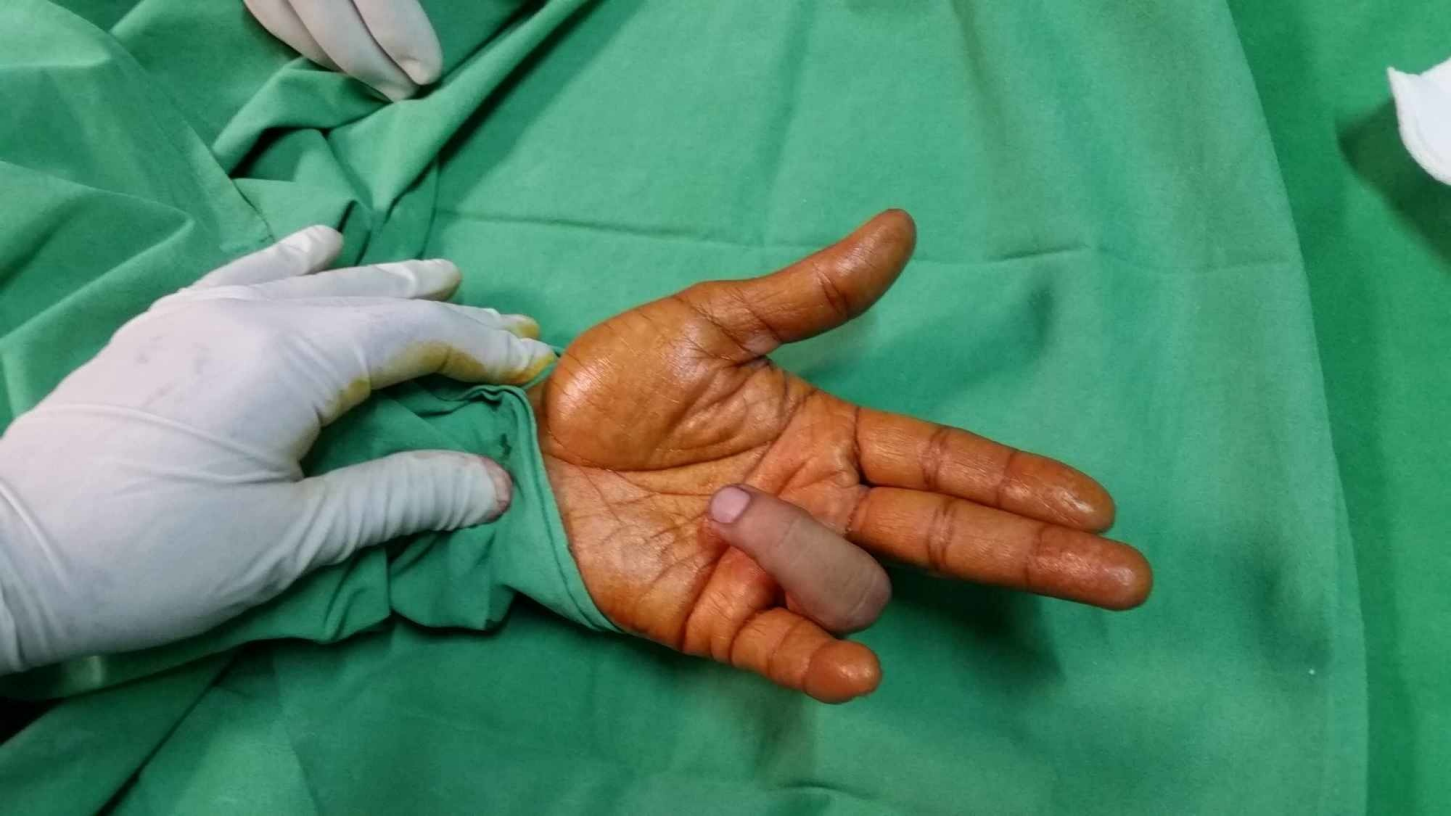
Preoperative

The preoperative flexed position of the involved digit is shown in Figure 1.

**Figure 2:**
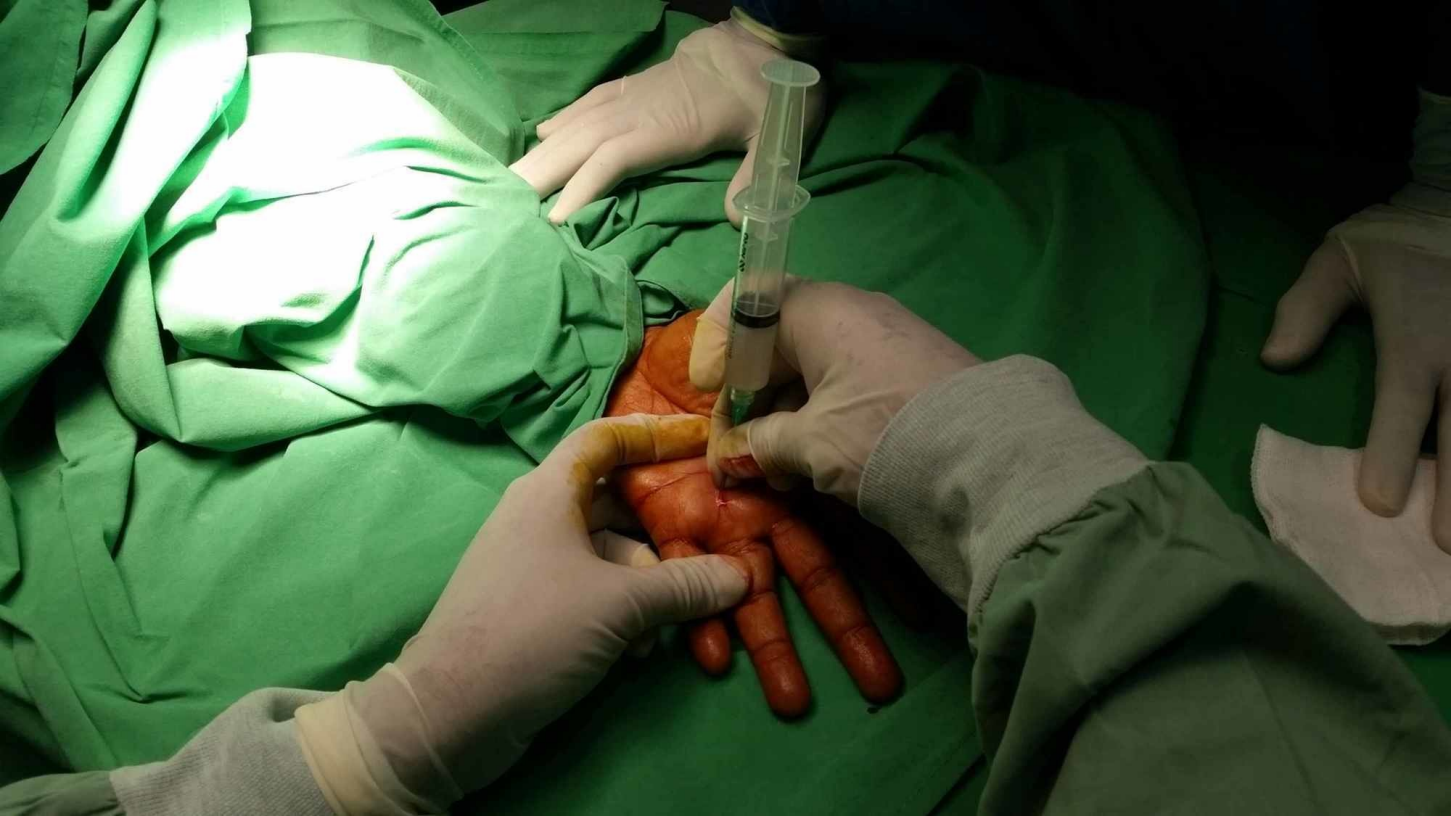
Percutaneous trigger digit release

The procedure is shown in Figure 2.

## Discussion

In light of the findings of this study, percutaneous release of trigger finger is a safe and reliable procedure. Pain was a common complication, which was more common in diabetic individuals. However, no other serious complication, including neurovascular damage, was reported.

This study has contributed to the safety and reliability of percutaneous release in trigger finger in both diabetic and non-diabetic patients. However, this study has its limitations. Patients with recurrent TF were excluded; they might have had a worse outcome. It was a single-center study with a smaller sample and a short study duration. Patients were followed for six months only, hence, long-term complications/outcomes cannot be assessed. 

As discussed earlier, individuals with diabetes mellitus have a higher frequency of TF. DM has been established as a bad prognostic factor for medical and surgical interventions in trigger finger [13-14]. Percutaneous release has been reported to be a safe procedure with no to minimum neurovascular damage and very few incidences of infection. In a study with the long-term follow-up of patients undergoing percutaneous release of TF, no long-term complication was seen, other than pain [1]. Similarly, in another study from Pakistan, the three-month outcome for the percutaneous release of TF was satisfactory in 90% patients and recurrence was seen in none [5].

Huang et al. conducted a longitudinal study with a large sample size to compare the outcome of percutaneous release of TF in diabetic and non-diabetic persons [10]. Short-term pain was seen in 5% of diabetic and non-diabetic digits each. In the long-term, pain was seen in 25% of the digits of diabetic patients and in 14% of the digits of non-diabetic patients (p = 0.058). They also reported recurrent triggering in 15% of the diabetic patients and only in 5% of the non-diabetic patients (p = 0.013) [10]. In comparison, by the end of one month, 40% diabetic patients in our study reported pain as compared to none in the non-diabetic group. However, we did not find any case of recurrence, maybe because our patients were not followed for a longer time period. Eastwood et al. followed their patients for a mean of 13 months and did not observe any recurrence [11].

In another recent study with 39 patients of Grade 3 trigger fingers managed with percutaneous release, hypoesthesia was reported in seven patients at the end of the first postoperative year and in two patients at the end of the third postoperative year. At the end of the first postop year, one case of tendon rupture was also reported. Recurrence was seen in five patients at the end of the first postop year and in nine patients at the end of the third postop year. There were painful scars in two patients by the end of the first year and in none by the third year postop. They did not report any incidence of superficial wound infection [15]. To the best of our knowledge, this is the only study that reported hypoaesthesia as a postop complication in percutaneous trigger finger release.

## Conclusions

Percutaneous trigger finger release is a safe, reliable, time-saving, and cost-effective procedure for the management of trigger finger in both diabetic and non-diabetic patients. Pain and superficial wounds remain a more common complication in diabetic patients; however, neurovascular complications were not seen.
